# Dynamics of Parental Opioid Use and Children's Health and Well-Being: An Integrative Systems Mapping Approach

**DOI:** 10.3389/fpsyg.2021.687641

**Published:** 2021-06-29

**Authors:** Jessica C. Smith, Leigh Alderman, Brandon K. Attell, Wendy Avila Rodriguez, Jana Covington, Brigitte Manteuffel, Ann M. DiGirolamo, Susan M. Snyder, Karen Minyard

**Affiliations:** ^1^Georgia Health Policy Center, Andrew Young School of Policy Studies, Georgia State University, Atlanta, GA, United States; ^2^Department of Educational Policy Studies, College of Education & Human Development, Georgia State University, Atlanta, GA, United States; ^3^Mathematica, Princeton, NJ, United States; ^4^School of Social Work, Andrew Young School of Policy Studies, Georgia State University, Atlanta, GA, United States

**Keywords:** opioid use disorder, systems mapping, parenting, children's health, systems thinking, substance use

## Abstract

The seemingly intractable opioid epidemic compels researchers, the media, and families to better understand the causes and effects of this complex and evolving public health crisis. The effects of this crisis on people using opioids, maternal prenatal opioid exposure, and neonatal abstinence syndrome are well-documented, but less is known about the impact of caregivers' opioid use on children's health and well-being. One challenge to understanding the effects of parental opioid use disorder (OUD) on child and adolescent outcomes is the numerous interrelated pathways in which a child's health and well-being can be impacted. To better understand these dynamic relationships, we applied a systems mapping approach to visualize complex patterns and interactions between pathways and potential leverage points for interventions. Specifically, we developed a causal loop diagram system map to elucidate the complex and interconnected relationships between parental OUD, social determinants of health at the family and socio-environmental levels, family strengths, social supports, and possible adverse impacts on children's physical and mental health and risks for future substance misuse. The goals of this research are to (1) identify factors and dynamics that contribute to the relationship between parental OUD and children's health and well-being and (2) illustrate how systems mapping as a tool can aid in understanding the complex factors and dynamics of the system(s) that influence the well-being of children and their parents or primary caregivers.

## Introduction

The adverse physical and behavioral effects of the opioid crisis for adults with opioid use disorder (OUD) and their infants are well-documented, but less is known about the effects of a caregiver's opioid misuse on child and adolescent health and well-being (Peisch et al., 2018; Winstanley and Stover, 2019). One challenge to understanding the effects of parental OUD on child and adolescent health outcomes includes the numerous interrelated pathways by which parental OUD can affect a child. Yet, distinguishing the various ways parental OUD may impact a child's health and well-being is critical to identifying opportunities to intervene with treatment, prevention, and support strategies.

To better understand these relationships, systems thinking principles and tools can be utilized to help diverse groups of stakeholders build a shared picture of a complex issue, across what are often siloed, reinforced perspectives, and boundaries. This shared picture includes developing an agreed-upon definition of the issue, the systemic structure and elements at play, and how those elements potentially influence, and feed back into each other. Accordingly, systems mapping is a collaborative, visual tool that makes more explicit the complex relationships, interactions, and pathways contributing to the outcomes, potential upstream intervention points to increase desired outcomes, as well as potential unintended effects of interventions (Arnold and Wade, 2015; Manteuffel et al., 2019). In this way, systems maps can help identify the elements that contribute to parental use and misuse of opioids, risk factors for developing an OUD, factors that can sustain cycles of substance misuse, intervention points for breaking such cycles, and potential pathways leading to adverse outcomes for both parents and children.

In this study, we develop one type of systems map-a causal loop diagram-to display a high-level, holistic depiction of some of the interconnected relationships between OUD, parenting, social determinants of health (SDoH), family health and environment, and children's health and well-being. The goal of this study is to further the understanding of dynamics in this complex, boundary-spanning issue by (1) identifying factors that contribute to the relationship between parental OUD and children's health and well-being and (2) illustrating the systems mapping process as a tool to view the structure of the system(s) that may influence the health and well-being of children and their parents or primary caregivers. By making these structures and relationships more visible, we aim to identify leverage points in the structure where interventions may have an impact, make connections between existing research, and present where additional research can fill gaps and test new hypotheses.

Considering that many readers may be new to systems thinking in general, and that only a handful of studies have conducted systems mapping of the opioid epidemic in particular (Jalali et al., 2020), we begin by describing the basic components of systems thinking and systems mapping. We then detail the specific methodology applied to the development and refinement of the causal loop diagram that is the focus of the current study. Next, empirical evidence from the literature and themes from our discussions with subject matter experts are provided to credibly establish the various causal relationships depicted in the causal loop diagram. The paper concludes with a discussion of potential intervention opportunities, gaps in the evidence base and areas for future research, and the strengths and limitations of the causal loop diagram.

## Materials and Methods

### Systems Thinking and Systems Mapping

Health promotion, including the promotion of parent and family well-being, is complex, shaped by a range of health determinants that interact and influence each other in non-linear ways and are dependent on a variety of health promotion systems (Baugh Littlejohns et al., 2018). These systems include several health and social systems that are often separated by invisible boundaries that contain distinct, yet interrelated elements which can influence each other and create feedback loops that reinforce cycles in ways that are virtuous or vicious. Delays between some of these interactions and eventual impacts mean that some relationships can be overlooked and not factored into intervention considerations. As such, many have called for systems thinking to be applied to health promotion science (Baugh Littlejohns et al., 2018). Systems thinking tools, such as maps and simulation models, have been used to study and address a variety of seemingly intractable public health challenges, including mental health services delivery, childhood obesity prevention, tobacco control, the opioid epidemic generally, and regional health system transformation (Homer et al., 2016; Zimmerman et al., 2016; National Cancer Institute, 2017; Powell et al., 2017; Manteuffel et al., 2019).

Problem-solving using systems thinking involves identifying and characterizing often invisible interactions, feedback loops, and information delays among system elements (components or variables of a system) that, together, determine the behavior of the system(s), and ultimately health outcomes (Currie et al., 2018). Rather than analyzing system “elements” individually, systems thinkers synthesize the relationships within and between elements to understand how they come together to produce the outcome(s) of interest. For example, a systems approach to identify why a health system is experiencing a spike in medical errors would not focus on characteristics of the individual provider, but rather on the structure of the system that may be producing the outcome (e.g., financial structure that incentivizes seeing more patients in a day, vs. a structure that incentivizes quality of health outcomes) (Currie et al., 2018). In this way, a systems map can support more informed choices by expanding traditional siloed practices and mental models and identifying potential trade-offs and advantages of proposed interventions that may be cross-cutting within a system (Goodman, 2018).

Systems thinking is a particularly valuable approach in health promotion as it helps reframe poor or beneficial health outcomes away from the individual unit (person, family), to the broader system(s) at play that produce outcomes within certain populations. This is especially relevant when examining the contributors and impacts of OUD or substance use disorder (SUD) more broadly because it requires us to think about what factors may be driving and perpetuating cycles of substance use and adverse childhood experiences (ACEs)-not just how we can intervene or prevent misuse or harm at the individual level. Using this mindset, we can begin to think about shifting the system to improve outcomes for parents, caregivers, children, and our communities.

### Approach and Process

Using a systems map to illustrate the complex dynamics influencing parental OUD and child health and well-being was inspired by the Georgia Health Policy Center's (GHPC) earlier work applying systems thinking. GHPC has used systems thinking and mapping to address a variety of public health concerns, including childhood obesity, neonatal abstinence syndrome, and children's behavioral health in Georgia. Specific to this topic, the center previously developed a systems map to describe the elements contributing to—and perpetuating—the opioid epidemic (see Supplement 1). GHPC's original opioid systems map describes the pathways from opioid use and misuse to individual and potential intergenerational outcomes (Manteuffel et al., 2019). Specifically, the map describes pathways through which people move into and out of (as well as back into) prescription and illicit opioid use and misuse, alternative treatment with or without opioid prescriptions, incarceration, death, as well as treatment and paths to stabilized recovery. The map also includes hypothesized intergenerational effects from persons misusing opioids as contributors to ACEs of their children, and the feedback loop from these experiences (with a delay) to next generation opioid or other substance misuse, as well as the contextual contribution of SDoH. The call for research on the connection between parental OUD, parenting, and child health and well-being provided an excellent opportunity for GHPC to take a more focused approach in one area depicted in the earlier opioid systems map—potential intergenerational risks for OUD. Our interdisciplinary team of researchers with expertise in behavioral health (child and adult, including OUD and SUD), sociology, Health in All Policies, SDoH, and systems thinking collaborated to develop one type of systems map, a causal loop diagram, to explore these dynamics.

### What Is a Causal Loop Diagram? Why Is It Used? How Do You Read It?

Causal loop diagrams begin with asking why certain phenomena occur, what variables and relationships are involved, and where feedback mechanisms are located that might promote or interrupt the outcome(s) desired (Haraldsson, 2004). They are comprised of four components which, together, shift the focus from linear relationships to more realistic interdependent relationships that can help illustrate the behaviors of the system (Haraldsson, 2004): primary variables, arrows, feedback loops, and delays (Lannon, 2012).

1. Stakeholders identify and agree upon primary variables.
2. Arrows show relationships between variables and flow from cause (tail) to effect (arrowhead). An (S) label assigned to an arrow indicates the two connected variables change in the same direction, and an (O) label indicates the two connected variables change in the opposite direction. For example: (S): When X increases, Y increases; or when X decreases, Y decreases. (O): When X increases, Y decreases; or when X decreases, Y increases.
3. Feedback loops are created from interactions between variables (often, a focus for potential interventions). The directional relationship of variables creates two types of feedback loops: balancing and reinforcing. Balancing loops (B) attempt to bring things to and maintain them in a desired state, often referred to as stable or stubborn parts of the system. A change of a variable in one direction then counters the change of a related variable in the opposite direction. An oft-cited example in systems thinking literature is a thermostat regulating the temperature in a house (Haraldsson, 2004). Another is hunger and food consumption. As hunger increases, food consumption increases (S), which then decreases (O) our hunger. Reinforcing feedback loops (R) occur where a change in one direction creates change in the same direction, thereby compounding change in that direction (think of a snowball rolling downhill as an example of compounding growth). Reinforcing feedback loops are often referred to as virtuous or vicious cycles (Baugh Littlejohns et al., 2018). One common example for a reinforcing loop is a bank account: money is deposited into a savings account, the account then generates interest, the interest then increases (S) the amount of money in the savings account, and the higher bank account balance increases (S) the amount of interest earned, and so on (Lannon, 2012). A vicious cycle has the opposite worsening effect.
4. Delays occurring between interaction and outcome. All systems have delays, which can range from seconds to years, and cause fluctuations in systems. A delay occurs when an interaction between two variables takes more time to produce an outcome than the rest of the system. For example, it takes time (which can vary) between turning on a shower and for the water that flows to become hot (Haraldsson, 2004). Another example is the delay between a child's exposure to one or more ACEs, and later known potential outcomes to appear.

Two causal loop diagramming rules are important to note. First, because causal loop diagrams are intended to identify and help explain the direction, (S) or (O), of relationships between variables, the variables should represent quantities that can vary over time to allow for statements that an increase in one variable will increase or decrease a related variable (Kim, 1992). As such, the variables we include in our causal loop diagram are framed in terms of “quality of,” because quality can vary over time; otherwise, it would be difficult to quantify the relationship between the variables. Second, it is recommended to use a positive sense of the variable name when possible (e.g., increasing or decreasing well-being is clearer than increasing or decreasing illness) (Kim, 1992).

### Developing a Causal Loop Diagram Systems Map

Our causal loop diagram was developed through an integrative, multistage process. In preparation for the first stage, the team reviewed the original GHPC opioid systems map and participated in an interactive mapping session facilitated by an external systems mapping expert. In the mapping session, the team began by identifying variables that play a role in three key domains: (1) risk of developing an OUD, (2) parenting abilities, and (3) child health and well-being. We then focused on variables that appeared to interact with and connect across multiple domains. The challenge then became understanding and visualizing connections among each variable and capturing the progression of these relationships over time and across generations. By the end of the session, the team had developed several draft maps to illustrate the complex relationships between parental OUD, family health and environment, SDoH, and child health and well-being.

In stage two, the team conducted a supplemental review of existing gray and peer-reviewed literature to identify the extent to which the literature supported (or conflicted with) the proposed causal pathways in the draft maps, as well as where gaps in research on potential causal pathways remain. To review the impact of OUDs on parenting in the context of child health and well-being and SDoH, we searched the following electronic databases: Google Scholar, JSTOR, PubMed, and ScienceDirect. Keywords used were *opioid use disorder, substance use disorder, parenting, parenting stress, family health, family functioning, social determinants of health, health inequities, child welfare, child maltreatment, adverse childhood experiences, child and adolescent development, child and adolescent mental health*, and *child and adolescent well-being*. We also checked reference lists and articles which cited relevant works. The findings from this stage of the literature review were used to combine pieces of the draft maps into a single causal loop diagram.

To leverage the dialogue systems thinking promotes among stakeholders that are often siloed, in stage three, the team further tested and refined the map through an interactive session that convened a diverse group of external subject matter experts in OUD, child and adolescent development and well-being, and ACEs, as well as individuals involved in the treatment and implementation of interventions for populations with OUD. Participants included three members of leadership in state programs focused on addictive disease treatment, prevention, and coordination; and four academic researchers with subject area expertise in child development, child welfare and maltreatment, maternal substance use, and synthesizing research to promote effective treatment and prevention strategies. One of the subject matter experts is a person in recovery whose lived experience brought a critical perspective to the development of the map.

At the convening, members of our research team provided a brief overview of our systems mapping approach, including a review of causal loop diagrams and an explanation of the relationships and dynamics presented in the map. The subject matter experts were then asked to provide feedback on their interpretation of the map, the appropriateness of relationships, guidance on the placement of map elements, and what variables should be included or excluded from the map. Their input was critical to informing the next phase of our literature review, making further revisions to the map, and helping the research team frame the contributions of the map within the existing body of research.

## Results

### Causal Loop Diagram Systems Map

**Figure 1:**
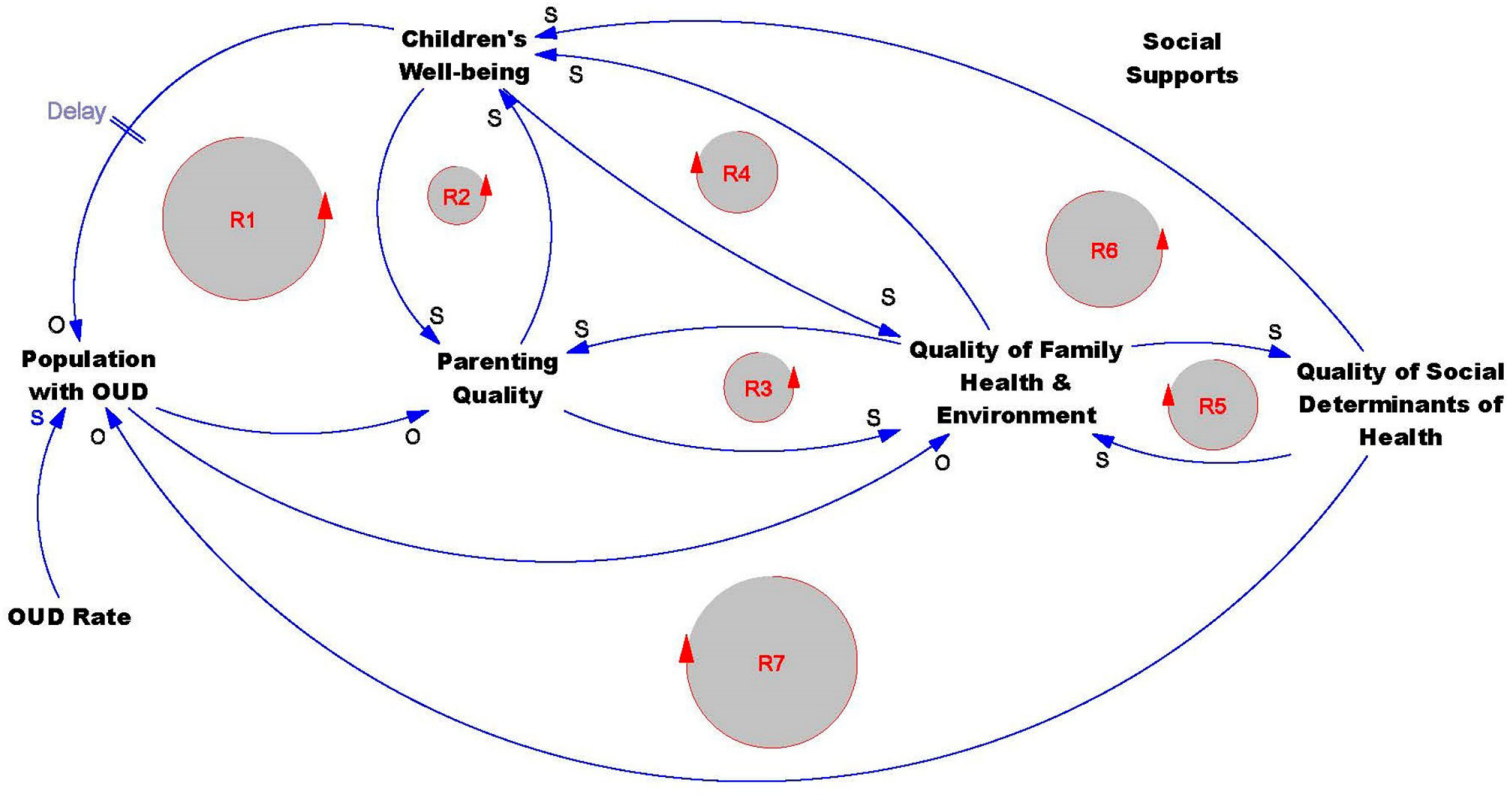
Casual Loop Diagram: Relationships between parental OUD and child well-being.

Our causal loop diagram and an interpretation of each map element is provided in Figure 1 and Table 1, respectively. In Figure 1, we identify primary variables, a series of unidirectional and bidirectional relationships between variables (represented by arrows), as well as reinforcing feedback loops that capture the mediation and interaction between multiple variables in the map. While we do not include any balancing loops in our casual loop diagram, we consider substance misuse as a balancing loop. In this loop, there is a physical need for a substance, taking the substance to meet this need returns the individual to physical equilibrium until the substance level attenuates in the body, triggering the cycle of use to begin again (Stringfellow, 2019). The causal loop diagram also includes one delay to represent a period of time between a state of health and well-being during childhood and risk of developing an OUD later in life. The relationships between elements portrayed in the causal loop diagram are supported and informed by findings in the literature and discussions with practitioners and researchers with expertise in child welfare, child development, and substance use treatment and prevention. We first address how input from subject matter experts was incorporated throughout our mapping process. Next, we discuss the mapped relationships and the literature corroborating the dynamics in the causal loop diagram.

**Table 1:** Casual loop diagram elements and definitions.

**Map element**	**Definition**
OUD rate	OUD rate is a measure of the prevalence of OUD. Specifically, it is the number of individuals diagnosed with an OUD per 100,000 individuals in the United States' population for a given year.
Population with OUD	A given OUD rate results in a population with OUD. Use of the term *population* acknowledges the varied social, economic, and demographic makeup of the individuals living with OUD and moreover recognizes that OUD is a social problem requiring numerous types of interventions (Salmond and Allread, 2019).
Quality of Children's Well-being	Child health and well-being is a multidimensional construct that encompasses the dynamic process of a child's physical, mental (cognitive, psychological), social, and material/economic situation as an outcome of intrapersonal, interpersonal, societal, and cultural processes (Pollard and Lee, 2003; Minkkinen, 2013).
Quality of Parenting	Parenting refers to support and promotion of a child's physical, emotional, social, and intellectual development with the goals of health and safety, preparation for life as a productive adult, and transmission of cultural values (Brooks, 2012; American Psychological Association (APA), 2021). Parenting is described by differing parenting styles, dimensions, skills, and practices (Smetana, 2017).
Quality of Family Health and Environment	Family health and environment refers to the physical and social conditions and climate of the family, including the health and well-being of family members, living situation, resources, structure and functioning, social dynamics and interactions within and outside the family. Vulnerable family environment (poor family functioning, low social support, and caregiver psychological distress) is an important predictor of children's mental health needs and functioning (Thompson et al., 2007).
Quality of Social Determinants of Health	SDOH refer to “the conditions in the environments where people are born, live, learn, work, play, worship, and age” (U.S. Department of Health Human Services, 2021) that impact health outcomes. SDOH can be grouped into five domains: economic stability; education access and quality; health care access and quality; neighborhood and the build environment; and the social and community context (U.S. Department of Health Human Services, 2021). These domains directly impact the experience of illness, the social patterning of population health and disease, and are recognized by many social scientists as the fundamental causes of disease and premature mortality (Link and Phelan, 1995; Phelan et al., 2004, 2010; Cockerham, 2013).
Social Supports	Social supports are broadly defined as the various types of help, aid, and assistance given by others that are perceived and/or received by an individual (Thoits, 2011).

### Subject Matter Expert Input

Feedback from our convening of experts was used to revise our initial causal loop diagram map and the focus of our literature review exploring the mapped relationships. The subject matter experts made significant suggestions and recommendations for changes in the causal loop diagram. Discussions on what the variables and elements of the map represented was an important part of our convening. While having a diverse group of experts involved in the discussion was desired to develop a map that aligned with systems thinking objectives, everyone came to the table with their own understanding and interpretations of what the terms used in the map meant. Once we were able to reconcile the varying perspectives, the subject matter experts involved in policy and programmatic decision-making encouraged the team to clearly define terms utilized in the map, which resulted in the creation of Table 1.

The subject matter experts also advised our team to include research that focused more broadly on SUDs, not just OUD. As a result, in the discussion of the map components, supported by the literature review, every relationship depicted in the causal loop diagram includes information on SUDs followed by specific information on OUD effects. Expanding the review to include other SUDs enhances the generalizability of the causal loop diagram and reminds us to consider how the mapped relationships also impact parents and families affected by other types of substance misuse. The subject matter experts also emphasized that children have varying needs throughout their life cycle and encouraged us to explore research on the effects of parental SUD and OUD on child health and well-being at different ages.

Based on our discussion with the subject matter experts, some substantial changes were made to the causal loop diagram presented at the convening (see Supplement 2). Changes to the map included adding an overarching social support component to capture the influence of social networks and supports on reducing stressors for parents, caregivers, and children; adding a feedback loop to illustrate the reinforcing relationship between SDoH on risk of developing an OUD; making the relationship between quality of parenting and family health bidirectional and adding a reinforcing loop; and making the relationship between parenting and child well-being bidirectional and adding a reinforcing loop. The causal loop diagram presented at the convening also included a variable representing “toxic stress,” but the subject matter experts recommended removing this from the map as it was difficult to define and risked oversimplifying the ways in which SDoH contribute to child well-being.

While we were not able to capture all subject matter expert viewpoints in our definitions and mapped relationships, reflections from the experts enabled us to revise our causal loop diagram to better represent both relationships identified in the literature and relationships observed in practice. Our hope is that the diversity of perspectives distilled in this map will make it relevant to a variety of stakeholders and researchers.

### Mapped Relationships

At the far left of the map, we begin with a stock *population with OUD*. In the causal loop diagram map the stock represents the accumulation of the population with OUD, which is driven by the prevalence of OUD (represented in the map as the element OUD rate). The remainder of the map's bolded components are the variables that each play an important role within the system. The map captures how the variables *parenting, family health and environment, social determinants of health*, and *children's well-being* connect with the stock population with OUD and the influence of *social supports* throughout the system. We incorporate evidence specific to parental OUD and more broadly parental SUDs to describe and support the causal loop diagram dynamics discussed in the remainder of this section.

#### Effects of Opioid Misuse on Parenting and Child Well-Being

To understand the ways in which parental opioid use can impact child well-being, we first examine how opioid use affects parenting. The impacts of parental OUD begin early. Newborns experience neonatal abstinence syndrome as an effect of maternal opioid or other substance use during pregnancy. Opioid use and medication-assisted treatment for OUDs during pregnancy can lead to neonatal abstinence syndrome or neonatal opioid withdrawal syndrome, specific to opioids, in some newborns (Substance Abuse and Mental Health Services Administration (SAMHSA), 2016). Infants with NAS often are born with low birth weights (Creanga et al., 2012; Patrick, 2015), may experience muscle rigidity, tremors, seizures, difficulty feeding, and be unable to regulate their core body temperature (Substance Abuse and Mental Health Services Administration (SAMHSA), 2016; Ko et al., 2017; Lynch et al., 2018). Infants prenatally exposed to opioids are often born pre-term and/or with low-birth weights, which may in turn contribute to a higher chance of developing long-term outcomes including cerebral palsy, developmental delays, and learning and behavioral problems (U.S. Department of Health and Human Services Health Resources and Services Administration (HRSA), 2014). However, known long-term outcomes of children exposed to opioids during pregnancy are still few and inconsistent (Sutter et al., 2014; Mactier and Hamilton, 2020).

Parental OUDs can also lead to an unstable relationship between parents and children and can be a predictor or consequence of child maltreatment and several maladaptive behavioral outcomes (Romanowicz et al., 2019). Parental OUDs can impair parents physically, emotionally, and mentally, which can compromise effective parenting. Parents' impaired emotional regulation can interfere with their responsiveness to child needs. This, in turn, can affect children's socioemotional development and later health outcomes. For example, young children of parents with OUDs show greater disorganized attachment (Mirick and Steenrod, 2016), and parental opioid use is associated with increased suicide risk among adolescents (Brent et al., 2019). Parental prioritization of substance use over a child's needs can lead to unsanitary and unsafe home environments or result in a parent's separation from their child due to incarceration (Davis and Shlafer, 2017) or the child being placed in foster care (Brook and McDonald, 2009; Testa and Smith, 2009; Berger et al., 2010). Children may also witness drug-related activity and be exposed to dangerous drug-related environments (Winstanley and Stover, 2019).

#### Dynamics Between Parenting and Child's Well-Being

The next step in the systems map is identifying how parenting directly influences child well-being among parents with OUD. Parent-child attachment plays a critical role in healthy infant development (Alhusen et al., 2013). During secure or healthy attachments, infants learn that they can rely on their caregivers for security. A caregiver fosters a secure attachment through responsiveness, which means that the caregiver pays attention to how the child moves and vocalizes, then the caregiver makes correct interpretations of when a child is tired, hungry, or sick, and quickly responds to provide consistent care that addresses the need the child is experiencing (Eshel et al., 2006). Responsive parenting can have protective effects on child development (i.e., increasing the quality of parenting increases child well-being). One of the main predictors of how well a child thrives is having at least one stable, consistent responsive adult in their lives. Responsive relationships early in life are important for building sturdy brain architecture and for providing the buffering protection needed to prevent challenging experiences from producing a toxic stress response and negatively affecting child outcomes (Center on the Developing Child at Harvard University, 2017). In the case of adolescent development, parent-child connectedness, authoritative parenting styles, open communication, and parental monitoring are shown to have a protective effect on adolescent high-risk behaviors (DeVore and Ginsburg, 2005).

However, parental substance misuse, such as opioid misuse, can distract parents from adequately responding to their children's physical and/or emotional needs (Smith et al., 2016). Enduring failure to meet a child's basic needs constitutes child neglect (Smith et al., 2016) and can result in an insecure attachment between parent and child. To date, the literature on parental OUDs and early childhood development has largely focused on mother-child dyads. A systematic literature review of 304 unique studies by Romanowicz et al. (2019), found that in direct observation studies, mothers with OUDs are more irritable, disinterested, ambivalent, and they also exhibit greater difficulty interpreting children's cues, resulting in their children developing insecure attachments. More information is needed on the father-child dyad and the effect of paternal OUD on child outcomes.

A child's well-being and behavior can also affect parenting and parental well-being. Parents' responsibilities include meeting their child's emotional and financial needs, ensuring the child's physical safety, and teaching the child how to have socially appropriate interactions with others. These activities, particularly if a child is experiencing challenges, can result in parental stress that can affect the general well-being and health of the parent, demanding emotional energy from them and potentially resulting in damaging effects on parents' attitudes and behaviors toward children (Jennings and Dietz, 2007). Generally, this type of stress is associated with a less positive outlook on parenting and less satisfaction in the parental role (Jennings and Dietz, 2007). In populations with SUDs/OUDs, this stress can increase parent's vulnerability to substance use (Rutherford and Mayes, 2019).

Financial and psychological difficulties associated with OUDs can also contribute to increased parenting stress (Suchman and Luthar, 2001), and/or a lack of social support (Luthar and Suchman, 2000). By their nature, OUDs can impair a parent's ability to maintain employment and increase the likelihood that a parent engages in illegal activities, which may worsen financial difficulties. Moreover, when a parent with an OUD also has a mental illness, the symptoms of the mental illness can exacerbate parenting stress, and work to diminish the attachment between a parent and child (Suchman and Luthar, 2001). Psychiatric medications may also contribute to further substance misuse due to competing effects of these medications and OUD treatment medications. Buprenorphine/naloxone, one OUD medication, has a negative effect on the dopaminergic circuitry, and serotonin reuptake inhibitors (e.g., paroxetine, fluoxetine, or fluvoxamine) may impede the body's metabolization of methadone and buprenorphine, which would trigger withdrawal symptoms and precipitate relapse (Snyder et al., 2019). Also, many people with OUD have other SUDs and engage in polysubstance use. Persons who use multiple substances tend to have worse mental health symptoms and are less compliant with treatment requirements (Snyder et al., 2019).

**Link to Mapped Relationships in Casual Loop Diagram**

Proposed causal relationships:

- Increased opioid misuse by a parent or caregiver can decrease the quality of parenting.
- Decreases in the quality of parenting can decrease child health and well-being.
- Declines in child health and well-being can decrease parenting capabilities.

Reinforcing relationships:

- The dynamics between opioid misuse, parenting, and child well-being are also captured by a reinforcing feedback loop in the causal loop diagram (R1). In R1, an increase or decrease in one section of the loop (population with OUD, quality of parenting, or child well-being) amplifies relational effects in the other loop variables. For example, a decline in a child's well-being due to parental opioid use and a decline in quality of parenting may increase the child's risk of later developing an OUD and, if they become a parent that misuses opioids, may decrease the quality of their parenting. This effect on the quality of parenting may in turn be a detriment to their child's well-being, perpetuating a negative cycle.
- In the causal loop diagram, reinforcing loop R2 illustrates that the relationship between parenting and children's well-being is not one-directional. Decreases in a child's well-being, whether due to physical, behavioral, or emotional matters, can create stress and challenges for parents and caretakers. Additional stressors may decrease the quality of parenting and lead to further declines in child well-being.

The causal loop diagram allows us to identify some of the distinct ways in which opioid use can negatively affect and decrease the quality of parenting and child well-being. These are some of the relationships that we may typically consider when thinking about the potential detrimental effects of parental OUD on children's health and well-being. Next, we begin to build upon these dynamics by introducing and connecting OUD, parenting, and child well-being to the other variables in our causal loop diagram.

#### Influence of Social Supports

In the causal loop diagram, social supports are presented as a variable but not connected to any other variable by causal arrows, warranting discussion of this important element of the map. Decades of previous research has strongly established the important role that social supports play as a mechanism that can ameliorate the impact of various adverse events on physical and mental health (Thoits, 1995, 2011; Uchino, 2006). At least three types of support are particularly salient: (1) Instrumental support, the oftentimes tangible help received in forms like financial assistance or daily help with routine tasks (Umberson et al., 2010); (2) Information support, advice and knowledge sharing that is received from others (Harvey and Alexander, 2012); and (3) Emotional support, the psychological help offered by others, for example encouragement and moral support offered during difficult times that individuals assign important meaning to (Semmer et al., 2008). Taken together, these elements of social support work to buffer against adverse outcomes of OUD.

Given the myriad protective effects that social supports provide and their presence throughout the literature that supports the elements and relationships in the causal loop diagram, we have not specifically mapped this variable to the others because doing so would render the causal loop diagram overly complex. It is possible that some may conceptualize social support as one type of a social determinant of health. We make the small but important distinction that social support networks, the linkages between the distinct set of individuals providing the various types of social support discussed above (Heaney and Israel, 2008), are more appropriately categorized as a social determinant of health, while social supports are more appropriately characterized as a mechanism through which variables act in the causal loop diagram.

Additionally, we make the distinction that these protective social support mechanisms can be conceptualized as prosocial social supports. Importantly, there are circumstances in which social supports can have unintended and even adverse consequences on physical and behavioral health outcomes (Rook, 1990; Dodge et al., 2006). For example, the breakdown of otherwise nurturing and supportive family bonds places youth and young adults at risk for becoming homeless, and once homeless these individuals may replace their family members with new social networks that can cause and reinforce a variety of maladaptive behaviors, such as substance use and risky sexual behavior (Wright et al., 2017). Additional research has found a similar relationship in youth exiting the juvenile justice system, who needed to limit their contact with negative peer influences to reduce the temptation to reengage with criminal activity (Martinez and Abrams, 2013). These types of negative influences are not only limited to youth and adolescents. For example, research among adults in recovery has found that avoiding potentially negative influences from others engaging in substance use was necessary to reach and maintain recovery (Weston et al., 2018; Pettersen et al., 2019). Therefore, our conceptualization of social support used in the current causal loop diagram can be thought of as the aspects of supportive relationships that help rather than harm.

#### Effects Between OUD, Parenting, and Family Health and Environment

Parenting skills and practices can directly affect family environments and how family members interact with one another within the larger social context (Moos, 1994; Greenberg et al., 2012). The effects of parental substance misuse on children can be viewed in relation to the family environment, and can manifest in detrimental effects on the physical, psychological, and cognitive functioning of the child (Kuppens et al., 2020). Parental SUDs are associated with lower levels of supervision, poor-quality parent-child interactions, and inconsistent discipline (Dunn et al., 2002; Arria et al., 2012). As a result, environments in which one or two parents or caregivers have an SUD are often characterized as traumatic and unpredictable, directly affecting the overall well-being of the family nucleus (Arria et al., 2012). Social norms within the home influence the environment and define the acceptability of drug use; children and adolescents who witness drug use or drug-related behaviors in their environment may perceive drug use as acceptable (Hawkins et al., 1992). Prior research shows that child involvement in parental substance use (i.e., opening an alcoholic beverage or lighting a parent's cigarette) is a predictor of child substance use (Bailey et al., 2018).

**Link to Mapped Relationships in Casual Loop Diagram**

Proposed causal relationships:

- Declines in the quality of parenting can decrease the quality of the family environment and health.
- Declines in the quality of the family environment and health can lead to decreases in the quality of parenting.
- Presence of OUD can decrease the quality of the family environment and health.

Reinforcing relationship:

- R3 in the causal loop diagram captures the reinforcing effects that parents and the family health and environment have with one another. For example, declines in the quality of a family's health and environment may generate stressors that further reduce parenting quality, which then feeds back into the dynamics at home leading to declines in family health.

#### Relationship Between Family Health and Environment and Children's Well-Being

While the interaction between parenting and family health and environment affects child ren's well-being, parenting itself does not mediate for all the dynamics between family health and environment and children's well-being. Separate from parenting, these two factors interact and influence one another. For example, household chaos, defined by disorganization or environmental confusion in the home and a variable of family health and environment influences children's well-being. Household chaos may include high levels of background stimulation, overly fast-paced family life, and lack of family routines, and is linked with caregiver education, family income, and the number of people living in the household (Marsh et al., 2020). Lower family income and higher number of individuals living in a household are correlated with higher household chaos, which is specifically related to adverse childhood outcomes including poor social-emotional functioning, cognitive development, academic achievement, and behavioral problems (Martin et al., 2012).

The effect of children's well-being on family health and environment can be seen in the relationship between children with disabilities and family health/environment. Children with disabilities may influence family health and environment positively by teaching family members positive characteristics. For example, siblings of children with Down or Rett Syndrome show positive personality traits including increased tolerance of difference, a compassionate nature, and increased maturity in comparison to their peers (Stoneman, 2005). Conversely, disadvantages to family health and environment also exist. Caregivers and families to children with disabilities report financial restraints on family outings, material goods, and other resources, as well as societal stigma and an overwhelming sense of household responsibilities (Dyke et al., 2009).

**Link to Mapped Relationships in Casual Loop Diagram**

Proposed causal relationships:

- Declines in the quality of children's well-being can lead to decreases in the quality of the family health and environment.
- Decreases in the quality of family health and environment can lead to declines in children's well-being.

Reinforcing relationship:

- R4 is a reinforcing loop connecting quality of family health and environment and children's well-being.

#### Relationships Between SDoH and Family Health and Environment

SDoH are the conditions within a home, family, school, and community that can impact a person's ability to be healthy and include factors like socioeconomic status, education, employment, social support networks, and neighborhood characteristics (Healthy People, 2020). When health inequities such as poverty, homelessness, or parental incarceration are present, the entire family is affected, not just the parent experiencing OUD (Chung et al., 2016).

Family health and environment are intertwined with and mediate the effects of SDoH on parental SUDs (Deatrick, 2017). Social networks, social supports, social cohesion, and social capital are important for the general physical and emotional well-being of individuals and communities. Social cohesion specifically refers to the sense of solidarity among members and social capital refers to the resources present in the community (Healthy People, 2020). For example, if a parent is unemployed, the other members of the family can assist with finances or they may have knowledge of existing job opportunities. Both resource and knowledge-sharing may lessen the effects of unemployment. By contrast, if this parent lived in an environment where the other family members were unemployed, or one had costly and recurring health-needs, the financial stress of unemployment would be more likely to severely affect the parent.

**Link to Mapped Relationships in Casual Loop Diagram**

Proposed causal relationships:

- Declines in the quality of SDoH can lead to decreases in the quality of the family environment and family health.
- Decreases in the quality of family health and environment can lead to declines in other SDoH.
- Both declines in quality of SDoH and family health and environment can exacerbate the effects of having a parent with OUD on parenting and child/adolescent outcomes.

Reinforcing relationship:

- R5 is a reinforcing loop connecting quality of family health and environment and SDoH. Declines in the quality of the family environment and family health can decrease SDoH. These decreases, in turn, can further lead to declines in other family health.
- Because quality of family health and environment shares a bidirectional relationship with children's well-being, quality of parenting, and quality of SDoH, a change in any of these variables will influence each remaining variable in the R3, R4, and R5 feedback loops, intensifying along the way. For example, increasing the quality of family health and environment can increase SDoH, which then can generate improvements in family dynamics that can improve the quality of parenting.

#### Connections Between SDoH and the Population With OUD

The literature highlights specific SDoH that are intertwined with and influence OUDs: incarceration, homelessness, and low socioeconomic status (Galea and Vlahov, 2002; Dube et al., 2003; Dasgupta et al., 2018; Barocas et al., 2019). Opioid use is higher in communities with high unemployment rates, and opioid overdoses are higher in communities with greater poverty and unemployment, and lower levels of education (Hollingsworth et al., 2017; Ghertner and Groves, 2018). High mortality rates as a result of an opioid overdose are also seen in populations that have just been released from incarceration. One study showed that the relative risk of opioid overdose death was 40 times higher within the first 2 weeks of release than that of the general population (Ranapurwala et al., 2018). Incarceration of a parent or caregiver can cause gaps in treatment and there may not be a smooth linkage to treatment, including the provision of medication-assisted treatment upon release.

Additional SDoH like access to healthcare and medical treatment, affordable housing, food insecurity, income inequality, structural racism, racial segregation, and stigma also influence opioid use and require further research to better understand the complexity of these relationships (Park et al., 2020). Research links substance use initiation via injection to specific neighborhood-level determinants such as income inequality, racial segregation, and low educational attainment (Fite et al., 2009; Friedman et al., 2016). Public health initiatives would benefit from further research in understanding the role and severity that each various determinant play on opioid initiation, sustained use, and recovery.

**Link to Mapped Relationships in Casual Loop Diagram**

Proposed causal relationship:

- Declines in the quality of SDoH can lead to an increased risk of OUD.

Reinforcing relationship:

- R7 connects the population with OUD, parenting, family health and environment, and SDoH. In this loop, a parental OUD can decrease the quality of parenting and family environment and health, which then feed into SDoH through (R3) and (R5). Declines in SDoH can then increase the risk of opioid misuse and further perpetuate the negative impacts this generates at other stages in the causal loop diagram.

#### SDoH Effects on Child Well-Being

Child well-being is also susceptible to the adverse effects of poor quality of SDoH. For example, poverty directly affects children's physical and cognitive development, as well as educational achievements and outcomes (Brooks-Gunn and Duncan, 1997). Poverty and low socioeconomic status are associated with higher risk of mortality in infancy and childhood, the onset of chronic illnesses, and are closely linked with child mental health problems (Spencer, 2003). Opportunities exist for SDoH to serve as protective factors for child well-being when one or both parents have a SUD. Healthy social support networks positively influence child and adolescent development. Connectedness, defined as a sense of being cared for, support, and a sense belonging, is a protective factor (Camara et al., 2017). Children and adolescents who feel a sense of connectedness are less likely to engage in high-risk behaviors such as substance use, sexual or criminal activity, and instead can produce an increased sense of autonomy, access to resources and health information, and engagement in social activities (Foster et al., 2017; Steiner et al., 2019). In-depth interviews with Black youth, ages 18-24, with at least one parent using substances, found that youth were less likely to engage in risky behaviors when they felt a sense of connectedness to other family members or loved ones. These relationships (e.g., uncles, aunts, grandparents) served as protective factors, highlighting the need for connectedness and nurturing relationships when there is an absence presented due to parental SUD or OUD (Offiong et al., 2020).

**Link to Mapped Relationships in Casual Loop Diagram**

Proposed causal relationship:

- Declines in quality of SDoH can decrease child well-being.
- Alternately, increases in SDoH such as increased social support networks can increase child well-being, even in an environment with parental opioid misuse.

Reinforcing relationship:

- R6 links the causal loop diagram variables parenting, family health and environment, SDoH, and child well-being. Because the relationship between each of these variables is the same (S), increasing (or decreasing) one variable in the loop perpetuates increases (or decreases) in the remaining elements.

#### Children's Well-Being and Risk of Developing OUD

Parental SUDs are linked to intergenerational substance misuse; having a parent with an SUD is a strong risk factor for the child or adolescent developing an SUD. Child and adolescent substance use are shown to be influenced by both genetic and environmental factors (Thatcher and Clark, 2008). A multisite longitudinal study on 295 children by Kaplow and colleagues found that lower levels of verbal parental reasoning and parental SUD are predictors of early-onset substance use for children (Kaplow et al., 2002).

Individuals with a higher number of ACEs are at greater risk for chronic disease, mental illness, violence and being a victim of violence (Felitti et al., 1998). Additionally, in studies of individuals with SUDs or OUDs, ACEs are often cited (Merrick et al., 2019). ACE scores range from zero to ten, with each type of trauma experienced by an individual counting as one point. In a study of 152 parenting women with OUDs, the total mean ACE score for the population was 4.3 (SD 2.3; range 0-8) and 65% of the sample reported having 4 or more ACEs, while only 5.0% reported zero ACEs (Gannon et al., 2020).

**Link to Mapped Relationships in Casual Loop Diagram**

Proposed causal relationship:

- A decline in child well-being can increase the risk of developing an OUD.
- An increase in ACEs and traumatic stress can lead to increases in the risk of developing an OUD.

Reinforcing relationship:

- The intergenerational risk of opioid misuse and reinforcing relationship between quality of parenting, child well-being, and developing an OUD is captured in R1.

## Discussion

### Opportunities for Intervention and Leverage Points

Developing a systems map allows researchers, practitioners, and policy makers to identify potential leverage points in the system where interventions may be effective in supporting a positive relationship or in modifying a more adverse relationship. For example, identifying parents at risk for substance misuse and providing parenting skills training and support as part of their treatment may be an effective way to change the relationship between parental OUD and poor parenting skills and consequential effects on poorer child well-being. SAMHSA reports that policies and procedures that encourage parents to enter substance use treatment and consider their parenting role as a part of their recovery process help to reduce the effects of parental SUDs on their children (Lipari and Van Horn, 2017). Helping parents to be more effective and nurturing with their children may also help to alleviate some of the stress that may lead to increased substance use.

Intervening in the relationship between the quality of parenting and child outcomes may be another potential leverage point where, in addition to parent skills training, identifying other adult caregivers in the home or in the nearby community (e.g., teacher, daycare provider, etc.) who can provide a stable, consistent positive environment for the child may help to buffer some of the adverse effects of having a less responsive parent due to opioid misuse. Another potential leverage point for disrupting the cycle of parental opioid use and adverse child outcomes may be in examining and addressing the various SDoH factors that impact parental opioid use and family environment such as economic stability, education access and quality, health care access and quality, and the communities in which people live, to identify families at risk and connect them with necessary social and health services to prevent or minimize some of these adverse behaviors and outcomes. Programs that address SDoH for families at risk may have broad reaching effects that can address multiple points in the causal loop diagram that may influence both parental opioid use and the effects of this on child and adolescent outcomes.

Laying out the various potential relationships in a systems map allows critical stakeholders to discuss where the most effective places may be to intervene, to collect data to further develop and refine how these factors work together to influence child outcomes and identify where the most effective upstream or downstream interventions may lie. This information can also then inform key policies to support parents who are struggling with substance use and their children.

### Strengths and Potential Limitations of the Study

Human capacity is limited in processing information reliably and accurately when that information involves elements that are interacting simultaneously (Bureš, 2017). Systems maps can help make such processes more explicit and understandable, while allowing others to share or surface their own mental models of those processes. These tools, however, do not remove the complexity of the system(s) at play, but can focus in on the parts of the system deemed relevant by those developing the map, by, for example, limiting which variables to include, or identifying the boundaries to apply in the map. A rule of thumb in systems mapping is that less is more, to start small and simple and add to the map iteratively as needed (Goodman, 2018). A more complex map may provide a more accurate representation of a system, but that accuracy gained in adding more elements and relationships may create the problem the mapping process is designed to avoid–creating a visual that is too complicated to comprehend (Bureš, 2017).

Our causal loop diagram is intended to serve as a new way to view and learn about the complex relationship between parental OUD and child health and well-being. It is in no way complete—it does not contain all variables, relationships, or feedback loops that factor into this relationship. With the causal loop diagram as a starting point, other researchers may seek to investigate, for example, what feedback loops are missing, or which variable dynamics are the strongest to recommend where to reinforce or interrupt specific feedback loops. Additional research can also identify the quantitative data needed to develop a more complex computerized systems dynamic models to test a variety of intervention options before implementing them (for example, see Jalali et al., 2021). As such, we have developed this map to catalyze new insights and dialogues among researchers and policymakers.

We also note that the literature review and discussion with subject matter experts that supported the causal loop diagram's development were not designed or intended to be an exhaustive look at all the complex dynamics between parental OUD and children's health and well-being. The map would have benefited from additional feedback from more parents and other individuals with lived experience, particularly to support its effectiveness as a tool to promote beneficial policies and programs. Because systems maps are often iterative, we may have an opportunity to incorporate perspectives from parents, families, and children in a future version of the casual loop diagram.

We recognize that while systems mapping is a powerful tool to visualize complex relationships in a simplified fashion, in the process of creating a map important relationships or variables will be omitted. For instance, the subject matter experts suggested that the map address impacts for both children and adolescents, and the current map does not differentiate this dynamic. However, we consider the input provided by subject matter experts to be a strength and valuable contribution to our understanding of the pathways that connect parental OUD to child health and development, what disparities exist that perpetuate intergenerational cycles of misuse, and where opportunities for change may exist.

### Gaps in Evidence, and Areas for Future Research

Subject matter experts who were interviewed mentioned comorbid mental health problems as significant factors related to OUDs, and this was supported in the literature. OUD is associated with comorbid psychiatric conditions, including major depressive disorder, which can also exacerbate OUD by making individuals with both conditions less likely to have psychological insight into their illness (Maremmani et al., 2007; David et al., 2008). Additionally, women, as opposed to men, are more likely to have comorbid mood or anxiety disorders (Evans et al., 2020). The scope of the current map does not explicitly capture this, although it could be considered as part of the quality of family health. This interaction and its consequences may be worth future exploration and could contribute to a better understanding of leverage points in the map. We also note that while the majority of the feedback provided by subject matter experts was directly supported by the existing literature, their recommendation to focus on protective factors for positive parenting and child well-being was challenging; the majority of the literature focuses on risk factors. Additionally, as mentioned in our results discussion, more information is needed on the effect of paternal OUD on child outcomes and the father-child dyad to balance what is known about the effects of maternal OUD on children's health.

We would be remiss to not acknowledge the impacts of the COVID-19 pandemic on substance use, parenting, and child well-being. At the time of our subject matter expert convening, the effects of COVID-19 were already being felt by some with SUDs/OUDs, and the experts asked if this was considered in the creation of the map. While new evidence and trends continue to emerge on the impacts of the COVID-19 pandemic on OUDs and the population, as well as treatment options, this was not an element emphasized in the causal loop diagram development. It will be important to remember parents with OUDs as the pandemic has exacerbated OUDs for some, and treatment has become more difficult to obtain. The loosening of some of the restrictions on the provision of treatment, including medication-assisted treatment that has occurred due to the pandemic may need to continue (Green et al., 2020). There has already been a call to revise and modernize addiction treatment services by improving access to care, including an increased use of telemedicine services, and providing care through more integration of specialists and non-specialists in response to the pandemic (Lopez-Pelayo et al., 2020). Additionally, social supports may have been impacted by COVID-19, and targeted interventions, including an increased use of technology, could be helpful (Weaver et al., 2020). These considerations are not yet presented in the map, however, moving forward should be more fully examined, particularly as they relate to parents.

## Conclusions

Using systems thinking, we developed a systems map to surface and understand the numerous, interdependent pathways by which parental OUD can impact children's health and well-being. Our aim was to: (1) create a visual map that captures the complex dynamics and elements that comprise the broader system of relationships between parental OUD and child outcomes and (2) illustrate how systems mapping can be applied to connect existing research and expertise across content areas to further our understanding this complex public health issue. Using systems thinking to address the challenge of parental opioid use and the lasting effects on children and adolescent outcomes can be an important tool in addressing the dynamic interplay among the various structures, systems and relationships involved, and in promoting critical, open dialogue around these issues.

## Data Availability Statement

The original contributions presented in the study are included in the article/Supplementary Material, further inquiries can be directed to the corresponding author/s.

## Author Contributions

JS organized and managed the research team, developed the research plan, participated in map development, subject matter expert convening, map revisions, and wrote and revised a significant portion of the manuscript. LA led the systems mapping development, revisions, and subject matter expert convening, also wrote sections on systems thinking and systems mapping. BA and BM contributed to research design, wrote portions of the results section, wrote definitions for system map variables, and revised the manuscript. BM also helped lead the convening of subject matter experts. WA and JC led the literature review phases and drafted a significant portion of the results section. SS participated as a subject matter expert at our convening, reviewed, revised, added to the initial literature review findings, and revised the manuscript. AD contributed to the research design, assisted in the subject matter expert convening, provided expertise in psychology, and contributed to the writing and revision of the manuscript. KM generated the idea for this manuscript, provided mentorship to the research team, assisted in the subject matter expert convening, and reviewed and revised the manuscript. All authors contributed to the article and approved the submitted version.

## Conflict of Interest

The authors declare that the research was conducted in the absence of any commercial or financial relationships that could be construed as a potential conflict of interest.
