# Supplementary figures and images for: Dynamics of Parental Opioid Use and Children's Health and Well-Being: An Integrative Systems Mapping Approach
Source: Front Psychol. 2021 Jun 29;12:687641. doi: 10.3389/fpsyg.2021.687641 (PMC8275850; doi:10.3389/fpsyg.2021.687641)

# Stock and Flow Opioid Systems Map

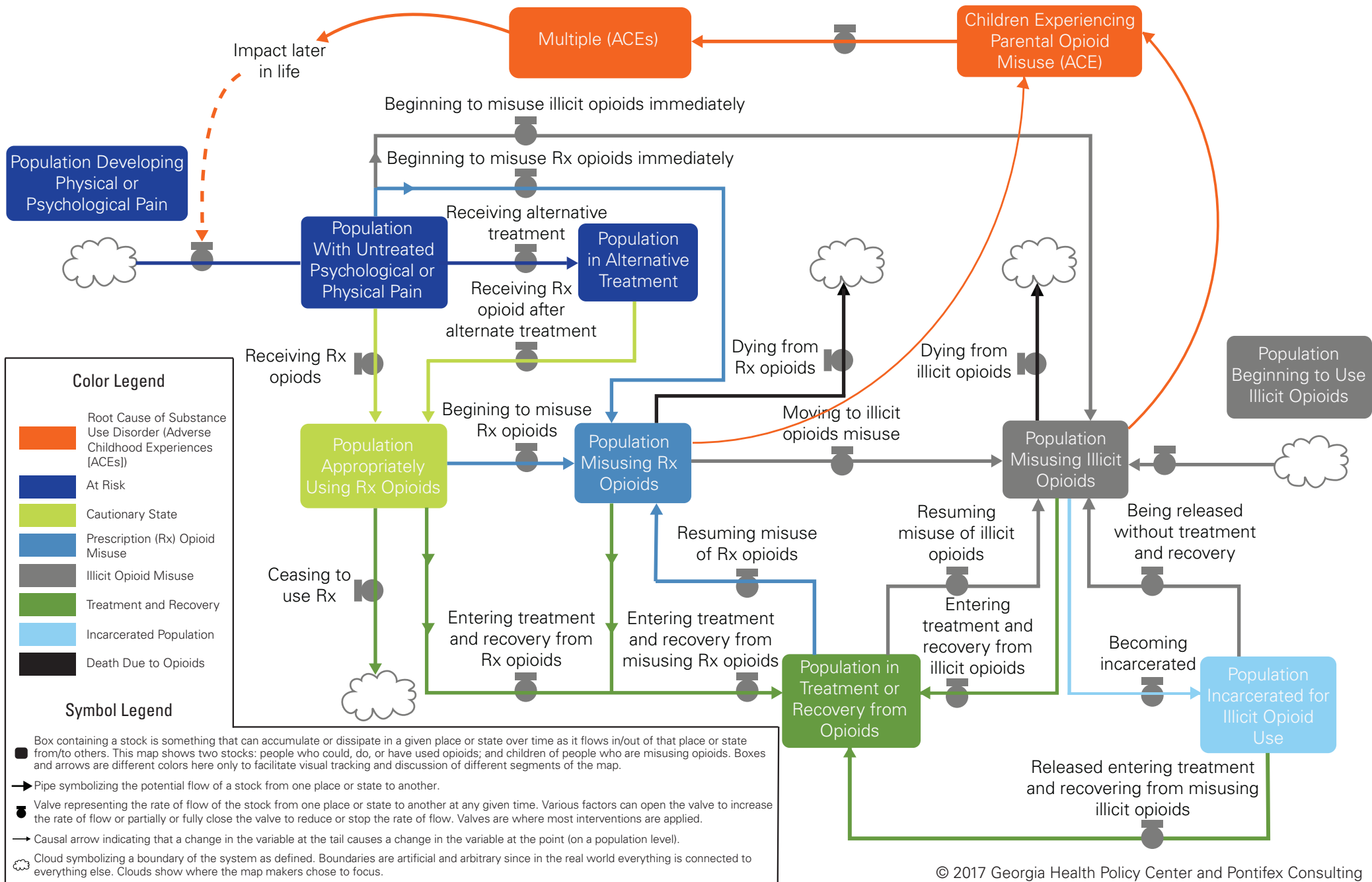

Supplement: Supplementary file 1 [file Data_Sheet_1.PDF]

**Supplement 2:** **Initial Causal Loop Diagram Shared with Subject Matter Experts**


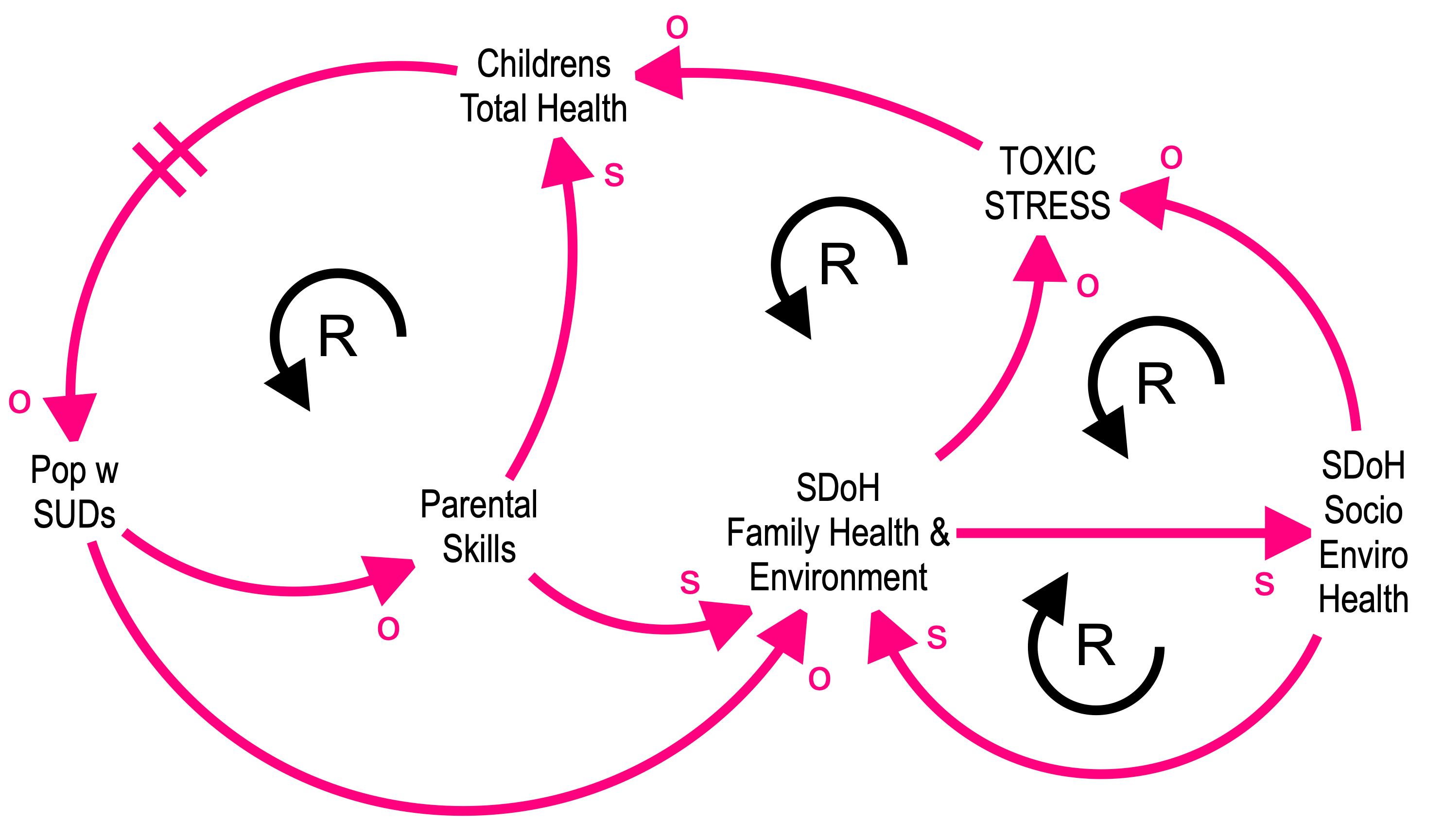

Supplement: Supplementary file 2 [file Data_Sheet_2.docx]
